# Protective Effect of *Eucalyptus radiata* Essential Oil-Based Nanoemulsion Against Pathogenic Bacteria and Spoilage Microorganisms on Fresh Beef Chunks

**DOI:** 10.3390/foods15132264

**Published:** 2026-06-24

**Authors:** Afranur Özçoban, Ayça Gedikoğlu

**Affiliations:** 1 Ministry of Agriculture and Forestry of the Republic of Türkiye, Üniversiteler Mah. Dumlupınar Bulvarı, No: 161, Çankaya 06800, Ankara, Turkey; afranurdnmez@gmail.com; 2 Department of Food Engineering, Faculty of Engineering and Architecture, Konya Food and Agriculture University, Melikşah Mah. Beyşehir Cd. No: 9, Meram 42080, Konya, Turkey

**Keywords:** aromatic oil, pectin, coating, antibacterial, foodborne pathogens, shelf life, red meat

## Abstract

The antimicrobial effect of *Eucalyptus radiata* essential oil nanoemulsion (EON) on *Staphylococcus aureus* and spoilage microorganisms was evaluated on fresh beef chunks during cold storage at days 0, 2, 4, 6, and 8. For this purpose, nanoemulsion was prepared using 2% eucalyptus oil combined with high methoxyl pectin, glycerol, and Tween 80, employing high shear force. Then the following were evaluated: (1) the essential oil’s chemical profile and in vitro antioxidant and antimicrobial capacities; (2) the nanoemulsion characteristics; and (3) the microbial counts of the beef treatments. The results showed that the essential oil’s primary components were o-cymene (45.4%), 2-bornene (26.29%), 1,8-cineole (11.31%), and α-pinene (9.25%). The EON had a particle size of 52.04 nm and a zeta potential of −9.16 mV. The in vitro studies revealed that both the essential oil and its nanoemulsion demonstrated significant antibacterial activity. Similarly, in in situ examinations, when the meat samples were spiked with *S. aureus* (0.1 × 10^8^ CFU/mL), the EON-treated meat samples had significantly (*p* ≤ 0.05) lower microbial counts than the untreated meat samples throughout the storage period; the difference between the treatments ranged between 1.62 and 2.44 log CFU/g. Additionally, the EON exhibited excellent antimicrobial efficacy against spoilage microorganisms on beef pieces during shelf life. On day 4, the maximum inhibitory activity was observed against total coliform, *Pseudomonas* spp., and yeast in reductions of 1.96, 2.09, and 2.18 log CFU/g in microbial counts, respectively. Moreover, application of meat samples with the EON delayed spoilage by 4 days. Therefore, the results of this study showed that coating beef chunks with the EON enhanced both product safety and shelf life.

## 1. Introduction

Essential oils are aromatic liquids obtained through the distillation process. They are extracted from plant components like leaves, stems, and flowers. Essential oils can have diverse chemical compositions with varying bioactivities, such as antibacterial, antifungal, antiviral, antioxidant, anti-inflammatory, etc. [1]. The class of chemicals called phenolic compounds is associated with the bioactivity of essential oils. The presence, concentration, and variation in phenolic compounds depend on many factors, including the kind of plant, which part of the plant is extracted, harvesting season, the climate, location, and extraction methodology [1,2,3]. In numerous studies, plant extracts and essential oils that are rich in phenolic compounds have demonstrated potent antimicrobial and antioxidant activity [2,4,5,6,7]. In the food sector, researchers have been investigating natural food preservatives that protect against pathogenic microbes and food spoilage and also reduce the use of synthetic preservatives in food processing. Therefore, utilizing essential oils as a mild preservation strategy in the food industry has received a lot of attention [7].

*Eucalyptus radiata*, also known as narrow-leaved mint, is one of the most widely used aromatic plants and is frequently preferred for its pleasant, rich minty scent [8]. Because of its antibacterial, antifungal, antioxidant, and anti-inflammatory properties, it is mostly used in the pharmaceutical and cosmetic industries [9]. In the food processing industry, eucalyptus oil is primarily used as a fragrance ingredient in the production of candies and chewing gum. Nevertheless, there has been little evidence of eucalyptus oil being used as a preservative in food products [10,11]. Owing to their potent bioactivities, essential oils (like eucalyptus oil) can be an alternative to synthetic additives to prevent food-related spoilage and ensure food safety [12]. However, it is challenging to use essential oils in the food industry due to their hydrophobicity, strong aroma, and volatile nature. It is necessary to introduce essential oils in suitable mechanisms that maintain their effects without compromising the food’s quality or sensory aspects. For this purpose, essential oils can be utilized in emulsions [13]. In their simplest form, emulsions are liquid–liquid dispersions that can be either oil in water or water in oil. The size of the droplets determines the classification of macroemulsions, microemulsions, and nanoemulsions. While all three emulsion types offer comparable advantages, like masking strong odors, preventing the loss of volatile bioactive compounds during the encapsulation process, and being easily applied through dipping or spraying, nanoemulsions are superior to the other two with additional benefits such as exhibiting less aggregation, better kinetic stability [14] and higher bioactivity [5].

These advantages have led to the effective application of essential oil nanoemulsions, including eucalyptus oil, in a variety of disciplines. For instance, Saranya Sugumar & Chandrasekaran [15] evaluated the wound management potential of a chitosan-based film impregnated with the eucalyptus oil nanoemulsion. The film was found to have significant antibacterial activity against *Staphylococcus aureus*. Additionally, several studies have shown the insecticidal potential of *Eucalyptus globulus* essential oil nanoemulsions [16] and nanogels [17]. There were also reports of antifungal and antibiofilm activity of *E. globulus* nanoemulsion against infection-causing fungi, *Candida* [18], and inhibitory activity against foodborne pathogenic bacteria [19]. Although in vitro research has yielded a wealth of knowledge, little is known about the potential of *Eucalyptus radiata* essential oil nanoemulsion in food applications. Therefore, the objective of this study was first to determine the in vitro antimicrobial and antioxidant properties of both *Eucalyptus radiata* essential oil and its nanoemulsion, and subsequently to assess the antimicrobial activity of the *E. radiata* essential oil nanoemulsion (EON) as an edible coating on fresh beef chunks during eight days of refrigerated storage against a foodborne pathogen and food spoilage microorganisms.

## 2. Materials and Methods

### 2.1. Materials

Plant material was gathered from Tarsus, Mersin, Türkiye (September 2023). Sodium sulfate anhydrous and 2,2-diphenyl-1-picrylhydrazyl (DPPH) were purchased from Sigma-Aldrich (Darmstadt, Hesse, Germany). Peptone water; tryptic soy broth; Mueller–Hinton agar; Baird-Parker agar; egg yolk tellurite; potato dextrose agar; violet red bile (VRB) agar; *Pseudomonas* CFC/CN agar; de Man, Rogosa, and Sharpe (MRS) agar; Tween 80^®^; glycerol; and NaCl were obtained from Merck KgaA (Darmstadt, Hesse, Germany). Empty, sterile test discs and antibiotic discs were supplied by Oxoid (Basingstoke, Hampshire, UK). Bacterial cultures were provided from the stock cultures of the Microbiology Laboratory of Konya Food and Agriculture University (Meram, Konya, Turkey).

### 2.2. Microwave Extraction

*Eucalyptus radiata* leaves were cleaned and dried until they reached a constant weight. Then, they were coarsely ground. For this procedure, methods from our previous study were slightly modified [4]. A sample was weighed and kept in distilled water (1:5, *w*/*v*) for 15 min. Then, the extraction procedure was conducted at 550 W for 45 min using an NEOS microwave extractor (MA 125 Milestone, Lombardy, Italy). Next, collected eucalyptus oil was put into a flask, and anhydrous Na_2_SO_4_ was added to the flask to remove residual water, after which the eucalyptus oil was transferred to a new vial. Finally, the vials were tightly sealed and maintained at 4 °C for further analysis.

### 2.3. Gas Chromatography–Mass Spectrometry (GC-MS)

To identify the chemical components in the eucalyptus oil, the method of Gedikoğlu et al. [4] was used. Two separate replications were carried out for this analysis.

### 2.4. Preparation of Nanoemulsion

The nanoemulsion of eucalyptus essential oil was prepared according to Gedikoğlu & Çıkrıkcı Erünsal [5]. To begin, 1% high methoxyl form of pectin was liquefied in sterile Milli-Q^®^ (Merck KgaA, Darmstadt, Hesse, Germany) water at 90 °C with constant mixing. Once the temperature of the pectin solution dropped to 25 °C, 2% eucalyptus essential oil, 5% glycerol, and 5% Tween 80^®^ (East Yorkshire, UK) were added gradually. Following that, an O/W nanoemulsion was prepared using the high shear force of WiseTis^®^ Homogenizer HG-15D (DAIHAN Scientific Co., Gyeonggi-do, Republic of Korea) at 20,000 rpm for 8 min. Then, the emulsion was deaerated, deposited in sterile, dark-colored glass containers, and stored at 20–25 °C in darkness for further investigation.

#### 2.4.1. Particle Size and Zeta Potential

The droplet size of the eucalyptus essential oil–pectin nanoemulsion was determined using a Zetasizer NanoZS laser diffractometer (Malvern Instrument Ltd., Worcestershire, UK) at 633 nm that was equipped with a backscatter detector (173°) at 25 °C. Also, the surface electric charge of the emulsion was determined in the continuous phase via phase analysis light scattering with a Zetasizer NanoZS laser diffractometer (Malvern Instrument Ltd., Worcestershire, UK) [20].

#### 2.4.2. Viscosity and Whiteness Index (WI)

A Vibrio SV-10 viscometer (Saitama, Japan) was used to measure the viscosity of the nanoemulsion at 25 °C in triplicate. Gold sensor plates of the instrument provided vibration with oscillation at an amplitude below 1 mm at 30 Hz.

To determine the whiteness index (WI) of the nanoemulsion, first the *L* (black = 0 and white = 100), *a* (a+ = red and a− = green), and *b* (b+ = yellow and b− = blue) values were obtained using a Ser-Lab SL400 colorimeter (İstanbul, Turkey). Then, the following formula was used to calculate the WI [21]:

$$WI=100-\sqrt{{\left(100-L\right)}^{2}+(a^{2}+b^{2})}$$

### 2.5. DPPH Free Radical Scavenging Activity

The multi-plate method previously described by Gedikoğlu [22] was used for this analysis. To begin, each well was pipetted with 100 μL of methanol. Then, the first column of wells (except the last row) was filled with 100 μL of eucalyptus essential oil. With proper mixing, half of the volume was transferred to the next well, yielding a 50% dilution for the following well. The final row was left for the blank (i.e., methanol-DPPH radical). Then, 100 μL of 0.004% (*w*/*v*) DPPH methanolic solution was placed to all wells, and the multi well plate was kept at 25 °C in darkness for 30 min. Finally, the results were obtained at 517 nm using a Synergy H1 microplate reader BioTek (Agilent, Winooski, Vermont, USA). The below formula was utilized to determine the inhibitions (%) of the DPPH radical.

$$I(\%)=\frac{A_{blank}-A_{sample}}{A_{blank}}\times 100$$

*A_blank_*: absorbance of the methanol and DPPH radicals

*A_sample_*: absorbance of a methanolic sample with the DPPH radical

After the inhibition percentages were computed, the sample concentrations were plotted against them. Then, the equation obtained from the plot was used to determine the sample concentration that would provide 50% inhibition (IC_50_). The procedure was conducted in triplicate.

### 2.6. In Vitro Antimicrobial Activity

#### 2.6.1. Preparation of Cultures

Cultures of *Bacillus cereus* NRRL B3711, *Enterococcus faecalis* ATCC 29212, *Staphylococcus aureus* ATCC 9144, *Staphylococcus epidermidis* ATCC 12228, *Escherichia coli* ATCC 25922, *Salmonella* Enteritidis ATCC 13076, and *Salmonella* Typhimurium ATCC 14028 were used for the antimicrobial assays. The bacterial cultures were preserved at −80 °C in a tryptic soy broth-glycerol solution. To activate the cultures, a loop of cell suspensions was transferred to the tryptic soy broth individually for 24 h at 37 °C in two consecutive cycles. Then, the tubes were centrifuged at 1714× *g* for 10 min at ambient temperature. The pellets were washed and were then ready for further analysis.

#### 2.6.2. Antibacterial Activity Screening of Microwave-Extracted *Eucalyptus radiata* Essential Oil

Eucalyptus oil’s antimicrobial activity was assessed against foodborne pathogenic bacteria using a disc diffusion assay. The pellets of cell cultures were diluted with 0.9% NaCl solution, and the concentration of cultures was set to 1 × 10^8^ CFU/mL (0.5 McFarland number) using a densitometer (Novolab, Geraardsbergen, Belgium). Then, 100 μL of the bacterial suspensions was spread on Mueller–Hinton agar. After that, the sterile discs impregnated with 10 μL of eucalyptus oil were placed onto the inoculated agar. The plates were incubated at 35 °C for 18 to 24 h. Inhibition values were determined by measuring the diameter of the clear zone in mm excluding the diameter of the disc. The test was performed in duplicate.

#### 2.6.3. Measuring the Minimum Inhibitory Concentration (MIC) Values for *Eucalyptus radiata* Essential Oil

The previously reported method of Gedikoğlu & Çıkrıkcı Erünsal [5] was employed to determine the MIC values. For this purpose, sterile 96-well plates were used. First, 100 µL of tryptic soy broth was dispensed to each well. Next, the same amount of eucalyptus oil was dispensed into the first column of the wells. After that, half of the volume of a well was transferred to the next well, providing a 50% decrease in the oil concentration for the consecutive wells. The dilutions provided an oil concentration range of 0.091 to 46.5 mg/mL. This procedure was conducted until the 10th column, leaving the last two columns for positive (broth and organisms) and negative control (broth), respectively. As previously mentioned, fresh overnight cultures of seven bacteria were serially diluted to a final inoculum density of 1–2 × 10^6^ CFU/mL for each bacterium. Later, 100 µL of the microbial suspensions were pipetted to each well, other than the 12th (final) column. After incubating microplates at 37 °C for 18 to 24 h, the MIC values were determined. The lowest concentration of eucalyptus oil with no visible bacterial growth was used to define the MIC values.

#### 2.6.4. Time-Kill Test of the Eucalyptus Nanoemulsion

The technique outlined by Guerra-Rosas et al. [23] was employed to assess the in vitro antibacterial effects of the EON. For this assay, *Staphylococcus aureus* ATCC 9144 and *Escherichia coli* ATCC 25922 were selected. Freshly prepared overnight cultures of bacteria (1 × 10^8^ CFU/mL) were mixed with the EON and purified sterile water in a ratio of 1:1:9 (*v*/*v*/*v*), respectively. Next, a series of dilutions was prepared, using 100 µL of the bacterial cultures from those dilutions that were plated on Baird-Parker agar for *S. aureus* and violet red bile agar for *E. coli* at 5, 30, and 60 min intervals to determine the inactivation of the bacteria. Plates were incubated at 37 °C for 24 h, and colonies were counted.

### 2.7. In Situ Antimicrobial Activity of the Eucalyptus Nanoemulsion

To evaluate the antimicrobial activity of Eucalyptus nanoemulsion coating, beef chunks were contaminated with *Staphylococcus aureus*. In most small deli shops, unsold meat cuts are often further cut into smaller pieces before being sold. This process exposes the meat to possible food handler contamination and increases the risk of *S. aureus* growth. Moreover, a recent study has reported a high prevalence of *S. aureus* in red meat samples in Türkiye [24]. Therefore, *S. aureus* was selected as a pathogen of interest in this study.

#### 2.7.1. Preparation of the Bacterial Culture

An overnight culture of *S. aureus* ATCC 9144 was grown in tryptic soy broth at 37 °C for 24 h. Subsequently, the cell suspension was centrifuged at 1714× *g* for 10 min at ambient temperature. The precipitated cells were rinsed twice and diluted in 0.1% sterile peptone water (*w*/*v*). Next, a 10-fold series of dilutions was prepared to adjust the final inoculum concentration to 0.1 × 10^8^ CFU/mL.

#### 2.7.2. Preparation of the Meat Samples

Fresh beef chunks were bought from three deli meat shops to provide three independent replications on the day of analysis in Konya, Türkiye. All of the fresh beef chunk replications were weighed, and 10 g samples were prepared. Then, the samples within the replications were randomly separated into two groups. The first control group did not receive any treatment, whereas the second group was treated with a nanoemulsion. The nanoemulsion group was submerged for 10 min in the eucalyptus nanoemulsion before being removed. The samples were then taken out and put on sterile stainless steel trays, where they dried for 10 min. Next, the top surface of all the samples was inoculated with 0.1 × 10^8^ CFU/mL of *S. aureus* ATCC 9144 and kept for 10 min to allow the bacteria to attach to the meat’s surface. Later, all of the treatments and their replications were individually placed on sterile Petri dishes, labeled, parafilm-sealed, and kept at 4 °C for 8 d. The antimicrobial activity of the eucalyptus oil nanoemulsion coating against *S. aureus* ATCC 9144-contaminated beef chunks were examined at days 0, 2, 4, 6, and 8. Next, 10 g of the sample was homogenized with 90 mL of sterile peptone water for 2 min using a stomacher Auto Blending System LS-700A (BNF, Gyeonggi-do, Republic of Korea). Then, serial dilutions were prepared and spread on Baird-Parker agar. The plates were incubated at 37 °C for 48 h. The number of colonies was expressed as log CFU/g of the meat sample.

#### 2.7.3. Shelf-Life Testing

To determine the inhibitory effect of the eucalyptus nanoemulsion against spoilage microorganisms in fresh beef chunks, samples were tested for total coliform count, *Pseudomonas* spp. count, lactic acid bacteria count, and yeast and mold count at days 0, 2, 4, 6, and 8. First, the meat treatment was blended in sterile peptone water in a ratio of 1:9 (*w*/*v*), and 10-fold serial dilutions were prepared. Following that, 0.1 mL aliquots of the diluted samples were transferred to the appropriate agar media using a spread plating procedure. VRB and MRS agars were employed to count the coliform and lactic acid bacteria, and the plates were incubated at 35 °C for 48 h. *Pseudomonas* spp. were counted using *Pseudomonas* CFC/CN agar incubated at 30 °C for 48 h. Potato dextrose agar was employed for the yeast and mold counts, and the plates were maintained at 25 °C for 72 h. The colony count was represented as log CFU/g of the meat sample.

### 2.8. Statistical Analysis

The in vitro data were analyzed using a two-sample *t*-test in Stata IC 14 (Stata Corp., College Station, TX, USA). The data from the in situ experiments were analyzed using a two-way analysis of variance (two-way ANOVA). By employing a generalized linear mixed model for the statistical analysis, the treatments and storage time were designated as fixed effects, while the replications were designated as random effects. A Tukey multiple comparison test was employed to identify significant differences between the means of the same treatment at different storage times. Additionally, the least significant difference (LSD) was utilized to identify significant differences between the means of different treatments on the same day at the 5% significance level.

## 3. Results and Discussion

### 3.1. Chemical Composition of Eucalyptus radiata Essential Oil

The chemical profile of *Eucalyptus radiata* essential oil obtained by microwave extraction was determined using gas chromatography combined with mass spectrometry. A total of 53 compounds were identified, and only the chemical compounds with a relative peak area greater than 0.5% are listed in Table 1. According to the findings, the four aromatic hydrocarbons with the highest concentrations detected in the *E. radiata* essential oil from Tarsus, Türkiye, were o-cymene (45.4%), 2-bornene (26.29%), 1,8-cineole (11.31%), and α-pinene (9.25%). Bendaoud et al. [25] investigated the chemical composition of Tunisian *E. radiata* essential oil and discovered that the greatest concentrations were 1,8-cineole (69.53%), α-pinene (11.94%), and trans-pinocarveol (4.81%). Furthermore, Luis et al. [10] studied the composition of Australian *E. radiata* essential oil derived using hydro-distillation and reported that limonene (68.51%), α-terpineol (8.60%), and α-terpinyl acetate (6.07%) were the most abundant compounds among the 72 detected. In previous studies, 1,8-cineole (eucalyptol) has been reported as one of the abundant compounds in eucalyptus oils [10,11], which is consistent with our findings. Also, the o-cymene concentration was the highest we found in *E. radiata* essential oil in comparison to previously published studies. This difference could be associated with many factors such as location, climatic conditions, and extraction methodology. In our previous work, we found that the microwave extraction technique provided a higher content of chemical compounds in lavender oil [3] and thyme oil [4] compared to supercritical CO_2_ extraction and hydrodistillation, respectively. While, the extraction procedure could affect the presence and concentration of o-cymene (45.4%), it is more likely that genetic variation and geographical differences caused such a high content.

### 3.2. DPPH Radical Scavenging Activity

The *E. radiata* essential oil extracted using a microwave method, which has a high content of o-cymene, 2-bornene, and 1,8-cineole, exhibited potent radical scavenging activity, with an IC_50_ value of 53.71 µg/mL. In previous studies varying antioxidant capacities of *Eucalyptus* species have been reported. For example, *E. radiata* from Australia exhibited a radical scavenging activity of 2.9 mL/mL, *E. globulus* from Spain showed 4.5 mL/mL [10], *E. camaldulensis* from Pakistan exhibited 89.11 µg/mL [26], and *E. robusta* from Australia showed 68.07 µg/mL [27]. Avila et al. [28] reported that molecules binding to the active sites of phenolic compounds, their positioning, and the availability of these active sites can alter their bioactivity. Singh et al. [29] examined the difference in the radical scavenging activity of the fresh and decaying leaves of *E. tereticornis* oil and found the IC_50_ values of 110 and 139.8 µg/mL, respectively. In a recent study, Park et al. [30] investigated the effect of different solvent ratios on *E. globus* essential oil DPPH radical scavenging activity and found that IC_50_ values were between 188.2 and 5841.7 μg/mL, and the best results were obtained from 30% ethanol extraction conditions. These studies demonstrate that various factors, such as plant variety, concentration of bioactive molecules, location, climate, and extraction methodology, influence antioxidant activity.

Furthermore, we obtained the IC_50_ value of 240.41 µg/mL for the eucalyptus nanoemulsion, which was lower than that of the pure essential oil. Contrary to our findings, previous studies [31,32] have found that essential oil nanoemulsions have stronger DPPH radical scavenging action than pure essential oil. Differences in the methodology, particle sizes, and formulation of emulsions could account for the variation in the results. However, the findings of this study suggest that the significantly lower antioxidant capacity can be attributed to the use of a small sample volume and the effect of oil droplet size variations resulting from an unstable emulsion with varying globule sizes (PDI = 1) on sampling.

### 3.3. Characterization of the Eucalyptus Nanoemulsion

**Figure 1:**
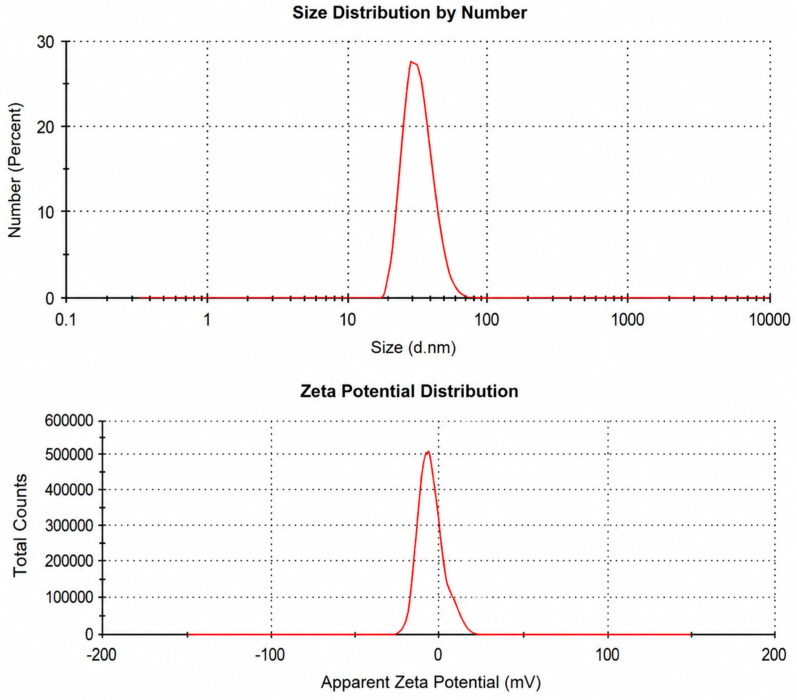
Droplet size and zeta potential of the *E. radiata* essential oil nanoemulsion.

To assess the characteristic features of the emulsion, the size, zeta potential, viscosity, and whiteness index values were assessed, with the results presented in Table 2. Emulsions are colloidal particulate systems in which the droplet size varies between 1 and 1000 nm. Depending on the particle size, emulsions can be classified as macro, micro, or nano. While nanoemulsions are thermodynamically unstable systems, their smaller particle sizes (1–100 nm) provide several advantages over emulsions. They have a higher dissolution capacity due to the inner oil phase allowing solubilization of lipophilic molecules, attaining a high capsulation rate, and acting quickly because they have a large interfacial area [33]. In this study, applying a high shear force and formulating the emulsion with 5% Tween 80^®^ resulted in a nanoscale particle size of 52.04 nm ± 31.44, which can be seen in Figure 1. Surfactants such as Tween 80^®^ are preferred owing to their low toxicity in comparison to ionic surfactants, being less sensitive to lower or higher pH values, and having a lower critical micelle concentration (CMC) [34,35]. Similar results were reported in previous studies using Tween 80^®^ as a surfactant [5,36,37]. It has also been shown that an increase in the concentration of surfactant (Tween 80^®^) has been inversely correlated with the particle size of the nanoemulsion [37]. Even though the nanoparticle size of 52.04 nm ± 31.44 using 5% Tween 80^®^ as a surfactant was easily attained, the high variability in the particle size (28 to 96.5 nm) and polydispersity index of 1 indicates that the nanoemulsion is unstable. On the other hand, [38] reported that surfactants such as Tween 20^®^ and Tween 80^®^ are prone to temperature dependent degradation. They found that the buffer of the surfactants kept at 25 °C and 40 °C had moderate and high oxidative degradation, respectively. This suggests that processing conditions such as temperature can affect surfactants and lead to unstable nanoemulsions. One of the key factors influencing the feasibility and consequently bioactivity of the emulsion is stability. Therefore, in future research, the use of varying surfactant concentrations and temperatures over a period of time should be examined to provide the most stable emulsion with the highest antimicrobial and antioxidant capacities.

The zeta potential of the eucalyptus nanoemulsion was −9.16 mV, as shown in Figure 1, which is important to note as the EON formulation affects the surface charge. The zeta potential is one of the key elements regulating the consistency of an emulsion by providing the net surface charge [39]. In most cases, electrostatic repulsions stabilize the aqueous emulsions. The particles with higher repulsive forces are less likely to agglomerate, resulting in a stable emulsion [40]. Aggregation occurs when the zeta potential is between −5 mV and +5 mV, whereas values exceeding ±30 mV produce a dispersed surplus of uniform-sized particles [41,42]. In our previous work, the *T. spicata* essential oil nanoemulsion was stable for only one week, with a low zeta potential of −2.95 mV [5]. In this study, we similarly obtained a low zeta potential of −9.16 mV. Although we did not observe agglomeration or phase separation visually in the nanoemulsion during the time of our studies, all of the findings, including particle size, PDI, and zeta potential, support that the nanoemulsion was unstable. In one study, investigators obtained an *E. globulus* essential oil nanoemulsion with a particle size of 76 nm, PDI of 0.22, and a low zeta potential of −9.42 mV [18]. In another study, Tween 80^®^ concentration at 4.63% was reported to be one of the most important parameters to improve the thymol nanoemulsion zeta potential and stability [43]. Moreover, Ullah et al. [44] found that increasing the homogenizer speed from 10,000 rpm to 18,000 rpm reduced the particle size from 561 to 130 nm and improved the zeta potential and stability of essential oil-loaded nanoemulsion. These studies show that many factors such as formulation and processing conditions have a significant effect on the characteristics of a nanoemulsion.

The EON can be described as an intermediate viscous emulsion with a viscosity of 82.67 mPa.s. This finding coincides with previous research [5,40,45]. Viscosity, one of the essential thermophysical properties of nanoemulsions, was influenced by many factors, including the type of biopolymer, presence of surfactant, variety of essential oil and its concentrations, and the techniques used to prepare the emulsion [20,40,45]. The biopolymer used in this study (i.e., pectin emulsion) was reported to have a viscosity of 39.2 mPa.s in our previous work [46], suggesting that the rise in the number of smaller droplets could boost the viscosity of the EON. In addition, the type of essential oil could influence the viscosity of the emulsion by affecting the absorption kinetics of the oil droplets in the continuous phase [47].

The *E. radiata* nanoemulsion prepared with a high shear force homogenizer provided a whiteness index (WI) value of 48.91. One study looked into the impact of microfluidization on the particle size and WI of essential oil emulsions. The thyme and lemongrass essential oil nanoemulsions possessed WI values of 49.6 and 47.9, respectively. When the emulsions were microfluidized, the particle sizes of the emulsions decreased to less than 20 nm. As a result, lower WI values were attained for the nanoemulsions; for instance, the lemongrass nanoemulsion had a WI of 35.71 [45]. Similarly, Guerra-Rosas et al. [40] reported a WI value of 32.94 for a thyme essential oil nanoemulsion with a particle size of 18 nm. These studies suggest that the smaller the particle size of the nanoemulsion, the lower the WI values are obtained. In addition, lower WI values are associated with better transparency, which is a vital attribute that can affect the acceptability of coated food products [48].

### 3.4. In Vitro Antimicrobial Activity of Eucalyptus radiata Essential Oil and Its Nanoemulsion

**Figure 2:**
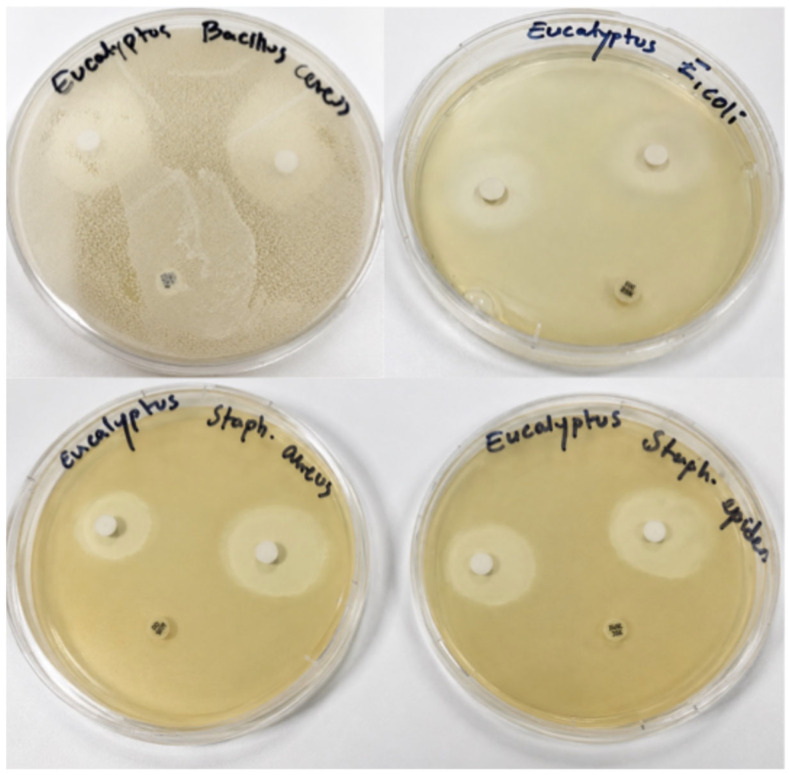
The inhibition zones of *Eucalyptus radiata* essential oil against the tested bacteria.

Table 3 shows the antibacterial activity assay findings for *E. radiata* essential oil and Figure 2 exhibits the inhibition zones of *E. radiata* essential oil against *B. cereus*, *Staphylococcus* spp. and *E. coli*. In the disc diffusion assay, the largest inhibition zones were found to be Gram-positive *Bacillus cereus* NRRL B3711 (24 mm) and *Staphylococcus epidermidis* ATCC 12228 (20.5 mm). However, the lowest antibacterial activity was seen against *Enterococcus faecalis* (5.5 mm) and *Salmonella* Typhimurium (4 mm). The essential oil of *E. radiata* extracted using a microwave showed a greater ability to inhibit the growth of Gram-positive bacteria compared to Gram-negative bacteria, which coincides with our previous research [4,5]. It is widely known that Gram-positive and Gram-negative bacteria have distinct cell structures and compositions. While Gram-positive bacteria miss an outer membrane, their cell wall structure includes a 20–80 nm thick peptidogylcan layer. In contrast, Gram-negative bacteria comprise three layers. The outer layer, which protects the cell, also sets them apart from Gram-positive bacteria. It consists of lipopolysaccharides and phospholipids. Furthermore, the outer layer holds proteins known as outer membrane proteins (OMPs), such as porins, that allow small molecules to pass through [49]. Gram-negative bacteria can increase their endurance to preservatives by modifying the hydrophobic properties of the outer membrane to block the diffusion pathways to hydrophobic molecules or by changing the porin structure to prevent the transfer of hydrophilic molecules [5,49]. This might clarify the reason for the enhanced effectiveness of eucalyptus essential oil against Gram-positive bacteria. Furthermore, as previously mentioned, numerous studies have demonstrated that essential oil’s antimicrobial potential is linked to biologically active chemicals (e.g., terpenes and terpenoids). The presence, concentration, variety of these active compounds, and their synergistic relationship can influence their antimicrobial activity. It was also found that the molecules that bind to the active sites of these compounds (as well as the molecules’ shape) play a crucial role in their antimicrobial properties [7]. Guimaraes et al. [50] observed that aromatic rings containing polar functional groups, such as oxygenated terpenes, displayed more potent antimicrobial effects in comparison to terpenes. The researchers discovered that oxygenated terpenes, like thymol, hinder enzymes in *S.* Typhimurium through their highly reactive hydroxyl groups, thus elucidating their mechanism of action. Our previous findings [4,5], support the conclusion that essential oils of *Thymus vulgaris* or *Thymbra spicata*, which have higher concentrations of oxygenated terpenes, such as thymol and carvacrol, exhibited greater antimicrobial activity (11 to 16 mm) against the same bacteria tested in comparison to *E. radiata* essential oil (4 mm), which is rich with monoterpenes such as o-cymene, 2-bornene, and 1,8-cineole (eucalyptol).

When the results of the broth dilution assay were examined, the MIC values were 11.63 mg/mL for all the tested Gram-positive bacteria and *E. coli*, but not for the Gram-negative *Salmonella* spp., which required a higher concentration of oil (23.25 mg/mL) for inhibition. Previous research examined the antibacterial activity of *Eucalyptus* spp. and their active compounds. Notably, 1,8-cineole and α-pinene demonstrated strong antimicrobial activity [51,52]. Furthermore, in a review study conducted by Balahbib et al. [53], the pharmacological characteristics of p-cymene were investigated, and it was discovered that p-cymene has high antibacterial, antifungal, and antiviral effects. While the position of active groups is known to alter the bioactivity of chemical compounds, such as para or ortho positions, the high antimicrobial activity of *E. radiata* essential oil could be associated with the presence of a high content of o-cymene, 1,8-cineole, and α-pinene in this study.

**Figure 3:**
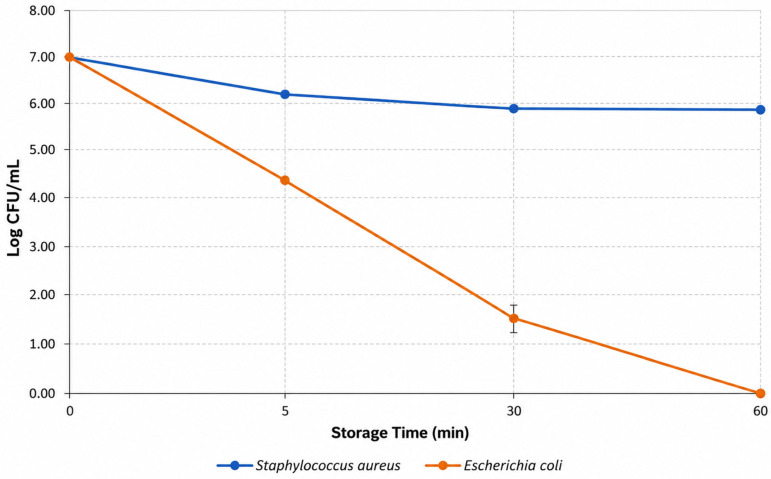
Antibacterial activity of the *E. radiata* essential oil nanoemulsion against Gram-positive and Gram-negative bacteria.

Figure 3 displays the time-kill assay results of the eucalyptus oil nanoemulsion (EON) against the tested bacteria. The assay measured the inhibitory effect of the EON individually with *S. aureus* and *E. coli* cultures in a water suspension at 5, 30, and 60 min. The EON provided a 1 log CFU/mL decrease in *S. aureus* and complete inhibition (7 log CFU/mL of reduction) in *E. coli* within the 60 min of the interaction period. Thus, the EON was substantially more efficient against the tested Gram-negative bacteria than Gram-positive bacteria. Furthermore, when the antibacterial activity of the eucalyptus oil and its nanoemulsion against Gram-positive and Gram-negative bacteria was compared, inverse relationships were noted. These findings are consistent with previous research with other essential oils and their nanoemulsions, such as thymbra oil [5], lemon grass and mandarin oil [23], sage oil [54], and thyme oil [55,56]. Despite the fact that the action mechanism is not well known, it has been shown that chemical components in essential oils, such as terpenes and terpenoids, react with the lipids in the bacterial membrane, disrupt the bacterial cell structure, cause leakage of cell material, and ultimately lead to cell death [57,58]. While this is true for both Gram-positive and Gram-negative bacteria, it is still not clear why the inhibitory effect of the essential oil increases against Gram-negative bacteria in nanoemulsion form. We predict that with the change in size and alteration in the hydrophilicity of essential oil nanoemulsions with surfactants, passing through porins will become easier. This can lead to an increase in the uptake of an antimicrobial nanoemulsion into Gram-negative bacteria cells over certain time periods, resulting in leakage and cell death.

### 3.5. In Situ Antimicrobial Activity of the Eucalyptus Nanoemulsion

#### 3.5.1. Survival of *Staphylococcus aureus* on Contaminated Fresh Beef Chunks

**Figure 4:**
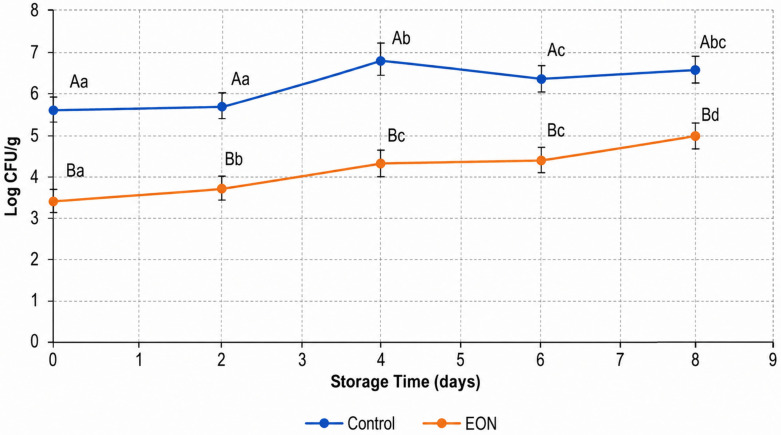
Effect of the *E. radiata* essential oil nanoemulsion (EON) coating on the inhibition of *S. aureus* ATCC 9144-contaminated fresh beef chunks. Different uppercase letters indicate significant (*p* ≤ 0.05) difference between two groups. Different lowercase letters indicate a significant (*p* ≤ 0.05) difference within the treatment at different storage times.

One of the aims of this study was to determine the antimicrobial efficacy of EON coating on *S. aureus*-contaminated beef chunks. For this purpose, beef chunks were contaminated with 10^6^ CFU/g of *S. aureus*. As seen in Figure 4, significant differences (*p* ≤ 0.05) were observed between the control and EON treatments. The initial *S. aureus* count of the control treatment was 5.72 log CFU/g, increasing to 6.61 log CFU/g on day 8 (i.e., the final day). In contrast, the microbial count in the EON group was significantly lower (*p* ≤ 0.05) compared to the control group; the count in the EON group was 3.44 log CFU/g on day 0 and reached 4.99 log CFU/g on the last day of storage. *S. aureus* counts increased in both groups throughout the storage period; however, the most significant difference between the groups was observed on day 4. The colony count in the control group was 6.78 log CFU/g, while in the EON group it was 4.34 log CFU/g, with the largest difference being 2.44 log CFU/g for all storage days. Additionally, the EON treatment reduced *S. aureus* counts by 2.56 log CFU/g on day 0 and showed strong inhibitory action against the bacteria. Later, the difference between the contamination level (6 log CFU/g) and counts of *S. aureus* in the EON treatments gradually decreased at days 2 (2.28 log CFU/g), 4 (1.66 log CFU/g), 6 (1.6 log CFU/g), and 8 (1.01 log CFU/g). Despite a gradual decline in antimicrobial activity, the EON application continued to demonstrate antimicrobial capacity against *S. aureus* throughout the shelf life of beef chunks. The decrease in antimicrobial activity could be due to the loss of volatile bioactive compounds over time. Even though the *E. radiata* level was maintained at 2% in the nanoemulsion for organoleptic purposes, the EON was an effective antimicrobial agent against *S. aureus*. Furthermore, compared to this study’s in vitro time-kill experiment, the EON showed greater antibacterial activity against *S. aureus* for meat application. Despite reports of the successful usage of essential oil emulsions during in vitro trials against *S. aureus* [5,59,60], only a few studies have investigated the effect of essential oil emulsions on the meat matrix. Moraes-Lovison et al. [61] studied oregano essential oil nanoemulsion-formulated chicken pate that was contaminated with 10^4^–10^7^ CFU/g of *S. aureus*. The use of an essential oil nanoemulsion in the pate was not effective against *S. aureus*. The authors suggested that the possible interaction of the phenolic compounds with the protein and fat in the pate caused a decrease in the antibacterial activity in comparison to their in vitro results. In another study, Almasi et al. In [39], it was found that biopolymer film made with a thyme oil microemulsion was able to decrease the *S. aureus* count by 1.4 log CFU/g in minced beef at the end of the storage period. These findings demonstrate how the antimicrobial agent’s efficacy is influenced by the food matrix and the delivery system. In addition, the EON coating achieves a high degree of success as a mild antimicrobial intervention technique in comparison to numerous other studies involving essential oils and emulsions.

#### 3.5.2. Results of the EON Application on Spoilage Microorganisms

**Figure 5:**
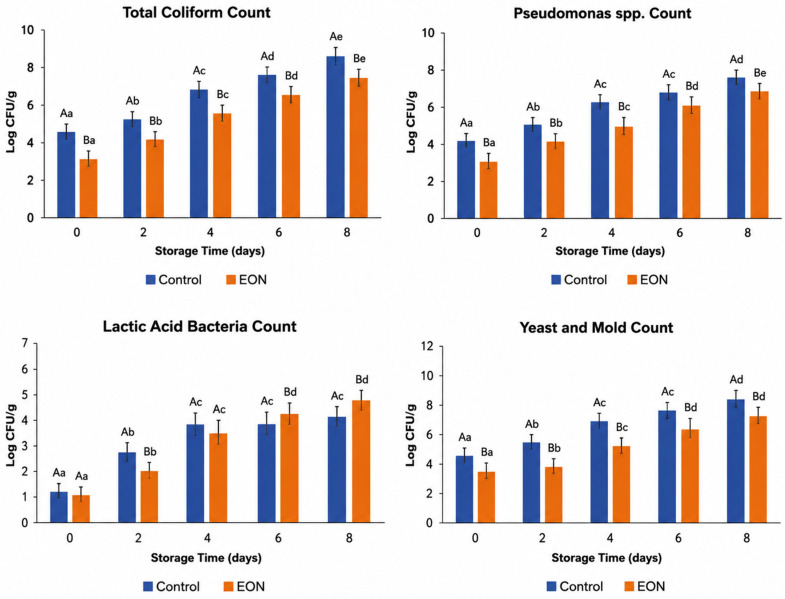
Effect of the *E. radiata* essential oil nanoemulsion (EON) coating on the spoilage microorganisms count of fresh beef chunks during cold storage. Different uppercase letters indicate a significant (*p* ≤ 0.05) difference between two groups. Different lowercase letters indicate a significant (*p* ≤ 0.05) difference within the treatment at different storage times.

Figure 5 displays the findings of the microbiological examination of fresh beef chunks kept in the refrigerator coated with an *E. radiata* essential oil nanoemulsion as an antimicrobial. Significant changes were seen between the control and EON samples for total coliform, Pseudomonas spp., yeast, and mold throughout the course of the 8-day storage period. The samples stored in the *E. radiata* nanoemulsion possessed strong antimicrobial activity against spoilage microorganisms. For the total coliform count, samples coated with the EON varied from 3.14 to 7.07 log CFU/g, while control samples ranged from 4.44 to 8.63 log CFU/g. The biggest significant difference was observed at day 4 between the treatments with 1.96 log CFU/g, providing a delay in spoilage for the EON treatment. Similar findings were reported by Xiong et al. [62], who found that an oregano essential oil resveratrol emulsion was an effective antimicrobial agent against the total viable count for fresh pork loin.

As presented in Figure 5, this study further shows the substantial antibacterial activity of an *E. radiata* essential oil nanoemulsion against *Pseudomonas* spp., with variations between the treatments ranging from 0.87 to 2.09 log CFU/g. Sonar et al. [63] discovered that the slow-release mechanism of oregano essential oil encapsulated with chitosan nanoparticles created an edible coating that was effective against psychrophilic bacteria on chicken patties during the storage period. In addition, Lu et al. [64] reported that 4% eucalyptus essential oil was effective in controlling *Pseudomonas* spp. on fresh pork.

While the EON exhibited strong antimicrobial activity against total coliform and *Pseudomonas* spp., it was not effective against lactic acid bacteria. The presence of lactic acid bacteria, when compared to other spoilage microorganisms, was substantially lower for both treatments. While the EON treatment had a significantly (*p* ≤ 0.05) lower lactic acid bacteria count at day 2, the opposite was noted for days 6 and 8. The results indicated that the EON treatment was not effective against lactic acid bacteria. Contrary to our findings, another study [46] found that an emulsion of thyme essential oil with a high thymol and carvacrol content was effective against lactic acid bacteria on sliced bolognas.

The EON treatment had considerably (*p* ≤ 0.05) lower yeast and mold counts during the 8 days of cold storage. Furthermore, compared to untreated control samples, the EON showed optimal antimicrobial activity against mold and yeast on day 4 (2.18 log CFU/g). Quatrin et al. [18] found in one investigation that a 5% *E. globulus* essential oil nanoemulsion had strong antibiofilm action against *Candida* spp. Although the store-bought beef chunks had a high initial microbial count for all the tested microorganisms, the control treatment reached the spoilage level at day 4, whereas the EON spoiled at day 8. The control samples exhibited a strong odor, a sticky and slightly slimy surface, and discoloration, whereas the EON treatment did not display these quality changes at the end of the storage day. As a result, the EON treatment improved the shelf life of fresh beef chunks by 4 days, effectively doubling their shelf life.

## 4. Conclusions

In the current study, 2% *Eucalyptus radiata* essential oil was employed with high methoxyl pectin to obtain an essential oil nanoemulsion. The high shear method was very effective in producing a particle size of 52.04 nm. Microwave-extracted *E. radiata* oil had a high content of o-cymene (45.4%), 2-bornene (26.29%), 1,8-cineole (11.31%), and α-pinene (9.25%) that could be associated with its high antimicrobial and antioxidant activity. Both the essential oil and its nanoemulsion demonstrated potent inhibitory effects on the tested pathogens during in vitro assays. The antibacterial activity of *E. radiata* essential oil in nanoemulsion form was notably enhanced against Gram-negative bacteria. The EON treatment provided full inhibition of *E. coli* in the time-kill assay after 60 min of exposure time in a water suspension. Furthermore, the *E. radiata* essential nanoemulsion was effective against controlling the growth of *S. aureus* both during in vitro and in situ conditions. Coating beef chunks with the EON significantly (*p* ≤ 0.05) reduced the number of *S. aureus* colonies, with the difference between the treatments ranging from 1.62 to 2.44 log CFU/g throughout the storage period. Moreover, the EON treatment negatively influenced the growth of spoilage microorganisms and delayed the spoilage of fresh beef chunks by 4 days. Promising results were obtained through both in vitro and in situ experiments; however, this study did not include the analysis of physicochemical parameters, such as pH and color measurements, as well as lipid and protein oxidation products, which also affect the shelf life of products. Future studies should examine the stability of emulsions related to changes in the formulation and processing conditions and their effect on quality attributes of meat products. Furthermore, consumer acceptance of meat products treated with eucalyptus essential oil nanoemulsion should also be investigated. Thus, the results of this study demonstrate that EON is a potent natural antimicrobial agent to improve food safety and shelf life.

## Figures and Tables

**Table 1:** Chemical composition of *E. radiata* essential oil obtained by microwave extraction.

No	Compound	Retention Time	Relative Peak Area (%)
1	α-Pinene	12.911	9.25 ± 0.01
2	β-Pinene	14.644	0.75 ± 0.01
3	Myrcene	15.095	0.98 ± 0.02
4	o-Cymene	17.338	45.4 ± 0.05
5	2-Bornene	17.525	26.29 ± 0.02
6	1,8-Cineole	17.610	11.31 ± 0.01
7	Neoalloocimene	32.736	1.37 ± 0.01
	Total peak area (%)		95.35%

Values are expressed as mean ± standard deviation (n = 2).

**Table 2:** Characterization of the *E. radiata* essential oil nanoemulsion.

Properties of Nanoemulsion	Values
Size (nm)	52.04 ± 31.44
Polydispersity Index	1
Zeta Potential (mV)	−9.16 ± 0.63
Viscosity (mPa.s)	82.67 ± 3.68
Whiteness Index (WI)	48.91 ± 1.24
DPPH (IC_50_, µg/mL)	240.41 ± 0.05

Values are expressed as mean ± standard deviation (n = 3). DPPH: 2,2-diphenyl-1-picrylhydrazyl, IC_50_: half of the maximal inhibitory concentration.

**Table 3:** Antibacterial activity of the microwave-extracted *E. radiata* essential oil.

Bacterial Species	Disc Diffusion Assay Inhibition Zone (mm)	Broth Dilution Assay MIC (mg/mL)
*Bacillus cereus* NRRL B3711	24 ± 2.00	11.63 ± 0.00
*Enterococcus faecalis* ATCC 29212	5.5 ± 0.50	11.63 ± 0.00
*Staphylococcus aureus* ATCC 9144	15 ± 0.50	11.63 ± 0.00
*Staphylococcus epidermidis* ATCC 12228	20.5 ± 0.50	11.63 ± 0.00
*Escherichia coli* ATCC 25922	15 ± 0.50	11.63 ± 0.00
*Salmonella* Enteritidis ATCC 13076	9.5 ± 0.50	23.25 ± 0.00
*Salmonella* Typhimurium ATCC 14028	4 ± 0.00	23.25 ± 0.00

Values are expressed as mean ± standard deviation (n = 2).

## Data Availability

The original contributions presented in this study are included in the article. Further inquiries can be directed to the corresponding author.
